# Systems perspectives on pediatric otitis media: environmental exposures, genetic susceptibility, and biomarker-guided interventions

**DOI:** 10.3389/fped.2026.1758098

**Published:** 2026-05-11

**Authors:** Ya Yu, Rui Yuan, Jingqi Zhang, Lu Wang, Tao Guo, Hanchao Shen, Hui Xie

**Affiliations:** 1 Chengdu University of Traditional Chinese Medicine, Chengdu, China; 2 Hospital of Chengdu University of Traditional Chinese Medicine, Chengdu, China

**Keywords:** environmental exposure, genetic predisposition to disease, host microbial interactions, microbiota, otitis media with effusion, otitis media, suppurative

## Abstract

**Background:**

Pediatric otitis media remains one of the most common childhood diseases worldwide and is a major cause of hearing impairment, recurrent healthcare utilization, and antibiotic exposure. Its pathogenesis is highly heterogeneous and can no longer be adequately explained by a traditional single-pathogen model.

**Objective:**

This review aims to synthesize current evidence on the roles of environmental exposures, host genetic susceptibility, biomarkers, and microbial factors in pediatric otitis media, with particular emphasis on their interactions and translational relevance.

**Content:**

Current evidence suggests that early-life environmental exposures may contribute to pediatric otitis media through convergent pathways involving epithelial dysfunction, impaired mucociliary clearance, inflammation, oxidative stress, and altered immune regulation, while host genetic variation may further modify susceptibility and disease heterogeneity. Emerging biomarker and microbial studies also support a systems-level understanding of pediatric otitis media and its therapeutic complexity.

**Conclusion:**

Pediatric otitis media should be understood as a multifactorial disorder arising from dynamic interactions among environmental exposures, host susceptibility, and microbial ecology. This integrated framework extends beyond pathogen-centered models and provides a conceptual basis for future biomarker validation, mechanistic stratification, and the development of more precise preventive and therapeutic strategies.

## Introduction

Otitis media (OM) is one of the most common infectious diseases in children (1), with particularly high prevalence in infants and toddlers (2). Epidemiological studies indicate that approximately 80% of children experience at least one episode of OM before the age of three, with the median age of first onset ranging from 6 to 12 months (3–5). In resource-limited settings, complications of OM often result in severe hearing impairment (6). Acute otitis media (AOM) has a high incidence and, in severe cases, can lead to serious complications such as acute mastoiditis, meningitis, and brain abscesses. Recurrent AOM or persistent otitis media with effusion may progress to chronic suppurative otitis media (CSOM) (7), which is now recognized as a leading cause of preventable hearing loss in both children and adults worldwide. In children, OM may cause temporary or permanent hearing loss, delayed speech and language development, frequent medical consultations, and substantial antibiotic exposure (8), while also being associated with impaired quality of life (9) and, in some studies, adverse cognitive and academic outcomes (10, 11). Globally, hearing impairment resulting from childhood OM has been identified by the World Health Organization (WHO) as one of the major causes of preventable hearing loss. This burden is especially pronounced in low- and middle-income countries, where it creates major barriers to education and imposes substantial socioeconomic costs (12). These findings underscore the urgent need for early risk identification and effective interventions for pediatric otitis media from a public health perspective.

The pathogenesis of pediatric OM results from a complex interplay of multiple factors, primarily involving intricate interactions among environmental exposures, host genetic susceptibility, and dysbiosis of the nasopharyngeal microbiota. In recent years, with advances in disease biology research, the traditional “single pathogen” model has gradually been replaced by an integrated etiological framework centered on the “environment–host–microbiome” triadic interaction (13). Within this new paradigm, the concept of precision medicine has been incorporated into the research and clinical management of pediatric OM, emphasizing the development of intervention strategies tailored to individual risk profiles (14). Notably, the advent of multi-omics technologies has facilitated the identification of molecular biomarkers and disease endotypes in pediatric OM, enabling risk stratification and targeted therapeutic interventions (15).

This review aims to systematically summarize current research on environmental exposures, genetic susceptibility, and dynamic biomarker changes in pediatric OM, with a focus on elucidating their interactive mechanisms and potential translational applications in precision interventions. The goal is to provide novel perspectives and theoretical foundations for the personalized management of pediatric OM.

## The association between environmental exposure and pediatric otitis media

Children exhibit distinctive anatomical, immunological, and respiratory developmental characteristics that increase their susceptibility to environmental pollutants. Consequently, they are more vulnerable to the onset and progression of otitis media. Epidemiological studies have consistently linked prolonged exposure to air pollutants—especially fine particulate matter (PM2.5), nitrogen dioxide (NO_2_), and ozone (O_3_)—with an elevated risk of pediatric otitis media. These pollutants contribute to upper respiratory tract mucosal inflammation, impair ciliary function, and predispose individuals to viral and bacterial infections, thereby facilitating middle ear infections. A large-scale cohort study in South Korea (16) demonstrated a significant positive association between long-term PM2.5 exposure and the incidence of AOM, sinusitis, and upper respiratory infections among children. Moreover, the frequency of outpatient visits for AOM increased proportionally with rising PM2.5 levels, reflecting a greater disease burden in pollution-exposed pediatric populations. Evidence from systematic reviews further supports an association between air pollution exposure and pediatric OM, although the strength and consistency of specific pollutant effects may vary across study designs and populations (17, 18). Diesel engine exhaust particles (DEPs), which contain polycyclic aromatic hydrocarbons (PAHs) and related compounds, have also been implicated in inflammatory airway and middle ear responses (19). A Polish birth cohort study demonstrated that prenatal PAH exposure was associated with increased respiratory symptom burden during the first year of life, suggesting that early-life pollutant exposure may contribute to airway vulnerability relevant to OM (20). Furthermore, Kim et al. (21) conducted a transcriptomic study using a mouse model and found that DEP exposure can cause changes in the expression of up to 697 genes in middle ear tissue, involving multiple signaling pathways related to inflammation, oxidative stress, and immune regulation. These findings support the biological plausibility that DEP exposure may contribute to OM pathogenesis through inflammatory and immune-modulatory mechanisms. Epidemiological data also indicate a strong association between early-life O_3_ exposure and increased risk of otitis media. Specifically, ozone exposure during critical developmental windows is statistically linked to both the onset and recurrence of otitis media, with stronger effects observed during periods of elevated temperatures or high pollution levels (22). A case-crossover study from Chongqing, China reported that short-term exposure to ambient air pollutants was associated with increased emergency visits for AOM among children. Among the pollutants, nitrogen dioxide (NO_2_) showed the most significant effect across all models, whereas the significant effect of ozone (O_3_) was only observed in seasonal stratification analyses. The study also identified children aged 0 years and 3–5 years as the most susceptible populations, with greater susceptibility during winter and spring (23).

Among indoor environmental factors, secondhand smoke exposure has garnered considerable attention for its impact on pediatric middle ear health (24). Evidence indicates that both prenatal and postnatal tobacco smoke exposure are associated with increased risk of OM in children. Constituents of tobacco smoke, including nicotine and other toxic combustion products, may impair immune function, alter mucociliary clearance, and disrupt Eustachian tube physiology, thereby facilitating ascending infection and recurrent disease (25, 26). Although some studies do not always distinguish exposure effects between children and adults, maternal–infant cohort studies and pediatric-specific investigations provide support for these associations.

Additionally, emerging evidence suggests that gut microbiota imbalance may indirectly influence susceptibility to otitis media through modulation of host immune responses. Recent studies have reported potential associations between gut microbiota composition and recurrent otitis media, supporting a possible gut–ear interaction, although current evidence remains limited and requires further validation (27, 28). In terms of immune susceptibility, allergic rhinitis (AR), a common immune-related disease in children, has also been recognized as a potential risk factor for otitis media (29). Some observational studies have shown that children with AR are at increased risk of otitis media, which may be associated with Th2-skewed immune responses (30), and that chronic mucosal inflammation and edema induced by allergens (e.g., pollen, dust mites, molds, etc.) may interfere with the function of the Eustachian tube, thereby increasing the likelihood of middle ear effusion (31, 32). Although most available studies are observational, these findings suggest that greater clinical awareness of comorbid AR may be warranted in children with recurrent OM or persistent middle ear effusion (30, 33, 34).

In social settings, frequent close contact following children's early entry into childcare significantly increases the risk of bacterial transmission. Studies have shown that children attending daycare centers carry common otopathogens more frequently than non-daycare controls; for example, nasopharyngeal carriage rates of *Streptococcus pneumoniae*, *Haemophilus influenzae*, and *Moraxella catarrhalis* were reported as 58% vs. 37%, 37% vs. 11%, and 80% vs. 48%, respectively, in daycare vs. control children. In addition, prospective data indicate that daycare attendance, siblings younger than 5 years, and nasopharyngeal colonization with major otopathogens are associated with increased risk of AOM in young children (35, 36). Furthermore, inadequate indoor ventilation may increase the burden of airborne pathogens, and indoor environmental conditions may also influence the risk of otitis media. Evidence from national cross-sectional data suggests that indoor exposures such as heating and household environmental factors are associated with otitis media in children (37).

In addition to the environmental factors discussed above, several related exposures and modifiers have also been implicated in susceptibility to pediatric OM. Seasonal viral epidemics and recurrent upper respiratory tract infections may further increase OM susceptibility by promoting immune activation, secondary bacterial co-infection, and shifts in the nasopharyngeal microbiota (23, 38). Breastfeeding appears to be protective against childhood AOM, likely through passive mucosal immune support and support of early immune maturation (39). These factors, together with the main environmental exposures discussed above, are summarized in Table 1.

**Table 1:** Key environmental exposures, mechanisms of action, and representative biomarkers associated with pediatric otitis media.

Key environmental factors	Type	Main mechanisms	Representative biomarkers	References
Air pollution (PM2.5, NO_2_, O_3_)	Physical/chemical stimulation	Epithelial barrier damage, NF-κB activation, inducing inflammation, increased oxidative stress	8-OHdG, MDA, IL-6, TNF-α	(17, 18, 22, 23)
Secondhand smoke exposure	Chemical toxin	Impaired mucociliary clearance, immune dysregulation, increased susceptibility to ascending infection	IL-8, IL-1β, CRP	(24–26)
Antibiotic overuse	Drug exposure	Altered maturation of nasal microbiota, reduced commensals, pathogen enrichment	↓ α-diversity, ↑ pathogen dominance	(98, 104)
Allergic rhinitis/allergic inflammation	Immune regulation	Th2-skewed inflammation, mucosal edema, Eustachian tube dysfunction	IL-4, IL-13, ECP	(30–34)
Close contact in childcare	Social behavior	Increased bacterial transmission, higher nasopharyngeal colonization with major otopathogens	*S. pneumoniae↑*, *H. influenzae↑*, *M. catarrhalis↑*	(35, 36)
Indoor environmental factors/poor ventilation	Environmental hygiene	Increased indoor exposure burden, altered household environmental conditions	Increased microbial burden/colonization density	(37)
Seasonal viral epidemics	Biological infection	Virus-induced immune activation, secondary bacterial co-infection, and nasopharyngeal microbiome alteration	IL-6, IFN-γ, nasopharyngeal viral nucleic acids	(23, 38)
Lack of breastfeeding	Nutritional factor	Reduced passive mucosal immunity and delayed immune maturation	↓sIgA, ↑bacterial adhesion rate	(39)

## Genetic susceptibility: from association to mechanism

### Evidence of familial clustering

Family clustering provides compelling evidence for the genetic susceptibility to OM. Genetic factors are estimated to account for approximately 40%–70% of the susceptibility to both AOM and otitis media with effusion (OME) (40). Single-nucleotide polymorphisms (SNPs) in immune-related genes such as *TLR4*, *IL10*, and *MUC5B* have been reported to be more prevalent among individuals with a family history of OM (41, 42).

Twin studies further substantiate the role of genetic predisposition. Monozygotic twins, who share 100% of their genetic material, consistently exhibit higher concordance rates for OM compared to dizygotic twins, who share approximately 50% (43). A prospective cohort study in the United States involving 168 monozygotic twin pairs and seven triplets estimated the heritability of OME to be as high as 73% by age two (43). Similarly, a longitudinal study of 1,373 twin pairs in England and Wales reported OM symptom heritability rates of 49%, 66%, and 71% at ages two, three, and four, respectively, with the influence of shared environmental factors diminishing over time (44). In a retrospective study from Norway comprising 4,247 twin pairs, structural equation modeling estimated genetic contributions to OM to be 72% in males and 61% in females (45).

In recent years, family-based studies and genome-wide association studies (GWAS) have identified multiple risk loci and candidate genes associated with OM, advancing understanding from familial aggregation to mechanistic genetic susceptibility. These genes fall into several biological domains, including immune regulation, ciliary structure and function, Eustachian tube and ear development, epithelial barrier integrity, oxidative stress responses, and microbial susceptibility. Immune-related genes such as *TLR4*, *TLR2*, *MBL2*, *TNF*, *IL6*, *IL10*, HLA antigens, and *NOD2* suggest that altered pathogen recognition, inflammatory signaling, antigen presentation, and mucosal defense contribute to recurrent or persistent OM (46–53). Genes involved in ciliary structure and mucociliary clearance, including *DNAH5*, *DNAI1*, *CCDC39*, and *CCDC40*, further support a role for impaired ciliary motility and abnormal axonemal organization in persistent middle ear effusion (54–57). Developmental and structural candidate genes also point to the importance of middle ear and Eustachian tube homeostasis in OM pathogenesis. GWAS findings have implicated *ANXA13* and *GAS2L2* in mucous or secretory OM (58), while *EYA4*, *TGFB1*, and *DYRK1A* suggest roles for developmental abnormalities, post-inflammatory remodeling, and Down syndrome–related susceptibility (59–62). Additional evidence implicates epithelial and oxidative stress–related genes, including *MUC5B*, *SOD2*, *GPX1*, *NFE2L2*, and *GSTM1*, as well as microbial susceptibility genes such as *A2ML1*, *FUT2*, and surfactant protein genes, in mucus retention, antioxidant defense, epithelial integrity, and host–microbiome interactions relevant to chronic disease risk (63–72). Representative candidate genes, their major biological functions, associated OM phenotypes, and proposed mechanisms are summarized in Table 2.

**Table 2:** Representative candidate genes associated with pediatric otitis media and their proposed biological functions and mechanisms.

	Gene(s)	Main function	Associated OM type(s)	Mechanism of action	References
Immune regulation	*TLR4*	Pathogen recognition, innate immune signaling	Childhood OM, severe phenotypes	Polymorphisms within the *TLR4* locus may alter pathogen sensing and downstream inflammatory signaling, thereby influencing OM susceptibility.	(50)
	*TLR2*	Pathogen recognition, inflammatory signaling	Recurrent AOM	Polymorphisms may affect early bacterial sensing and inflammatory responsiveness, contributing to recurrent disease risk.	(51)
	*MBL2*	Innate immune recognition	Recurrent OM, rhinovirus-associated AOM	Encodes mannose-binding lectin; functional variants may reduce complement-mediated clearance and increase OM burden in young children.	(46, 51, 52)
	*TNF*	Proinflammatory cytokine	Recurrent OM/OM-prone phenotype	Promoter polymorphisms may alter proinflammatory cytokine production and increase susceptibility to OM and tympanostomy tube placement.	(48)
	*IL-6, IL-10*	Proinflammatory cytokines	AOM, recurrent OM	Modulate inflammation; genetic variants may affect individual susceptibility.	(47)
	HLA antigens	Antigen presentation, adaptive immunity	Recurrent AOM	Variation in HLA-related antigen presentation may influence host susceptibility, although current evidence is older and less specific than that for innate immune genes.	(49)
	*NOD2*	Intracellular pathogen sensing, mucosal defense	OM (mechanistic relevance)	Contributes to NTHi-induced β-defensin 2 responses and mucosal defense in human middle ear epithelial cells.	(53)
Ciliary structure and function	*DNAH5*	Dynein motor protein, ciliary motility	OME, PCD-associated OM	Encodes a core component of motile cilia; dysfunction leads to impaired mucociliary clearance.	(54)
	*DNAI1*	Dynein intermediate chain	PCD-associated OM, chronic middle ear disease	Disruption of rhythmic ciliary motion impairs mucociliary clearance and middle ear fluid drainage.	(55)
	*CCDC39*, *CCDC40*	Ciliary microtubule assembly	OME, PCD-associated OM	Defects in axonemal organization and ciliary architecture impair motility and may promote persistent middle ear effusion.	(56, 57)
Eustachian tube and ear development	*ANXA13*, *GAS2L2*	Candidate genes associated with mucous/secretory OM	secretory OM/mucous OM	GWAS suggests association with mucous/secretory OM, but the precise functional role in middle ear homeostasis requires further clarification.	(58)
	*EYA4*	Ear and Eustachian tube development	OME/heritable OM	Deficiency disrupts middle ear cavity and Eustachian tube development, predisposing to otitis media with effusion.	(60)
	*TGFB1*	Cell growth regulation, tissue repair	OME, chronic OM	Regulates post-inflammatory repair and fibrosis; dysregulation promotes chronicity.	(59)
Chromosomal abnormalities	*DYRK1A*	Chromosome 21-associated protein kinase	Down syndrome–related OM	Increased *DYRK1A* dosage may contribute to frequent and severe otitis media in Down syndrome.	(61, 62)
Epithelial barrier function	*MUC5B*	Mucin regulation	OME/chronic OM	A major mucin component of middle ear effusions; altered expression may promote mucus retention and chronic secretory phenotypes.	(63)
Oxidative stress response	*SOD2*	Superoxide scavenging, antioxidant defense	Chronic OM	Eliminates mitochondrial superoxide radicals; protects mucosa from ROS damage	(64, 67, 79)
	*GPX1*	Hydrogen peroxide reduction	OME	Controls H_2_O_2_ levels; mitigates oxidative damage and inflammation	(66)
	*NFE2L2*	Antioxidant transcriptional regulation	OME, chronic OM	Activates expression of antioxidant response genes; alleviates oxidative stress and inflammation	(67)
	*GSTM1*	Glutathione transferase, ROS detoxification	Chronic OM	Null variants reduce ROS clearance capacity, increasing chronic inflammation risk.	(68)
Microbial susceptibility	*A2ML1*	Protease inhibition, epithelial defense	Recurrent and chronic OM	Encodes a protective barrier protein in the epithelium; mutations reduce resistance to exogenous proteases.	(69, 80)
	*SP-A*	Surfactant proteins, antimicrobial immunity	Recurrent OM	Contributes to innate immune defense in the middle ear and may influence susceptibility to recurrent OM.	(70)
	*SP-D*	surfactant-associated innate defense	OME	Expressed in the Eustachian tube and may contribute to mucosal defense and immune homeostasis.	(71)
	*FUT2*	Glycosylation, secretor status determinant	Recurrent OM	Non-secretor individuals lack certain antigens, altering mucosal colonization by pathogens such as *S. pneumoniae.*	(65, 72)

### Innate immune recognition and inflammatory regulation

Innate immune recognition and inflammatory regulation represent an important mechanistic link between host genetics and OM susceptibility. Human genetic studies suggest that variants in genes involved in pathogen sensing and early inflammatory signaling may influence disease risk, although the strength of association varies across cohorts. Polymorphisms within the *TLR4* locus have been associated with childhood OM in some populations, particularly in more severe phenotypes, although replication across independent cohorts has been incomplete (50). Likewise, *TLR2* polymorphisms have been linked to recurrent acute otitis media in early childhood (51). The *MBL2* gene encodes mannose-binding lectin (MBL), a key molecule in lectin pathway activation (46), and OM-specific studies further suggest that functional *MBL2* variants may increase OM burden in younger children and predispose to rhinovirus-associated AOM (51, 52). Together, these findings support the view that genetically determined variation in innate immune recognition may contribute to recurrent or persistent OM. Mechanistic studies further strengthen this interpretation. *NOD2*, an intracellular pattern-recognition receptor, contributes to nontypeable *Haemophilus influenzae* (NTHi)-induced β-defensin 2 responses and mucosal defense in human middle ear epithelial cells (53). In addition, recent reviews of host genetics in OM have consistently identified innate immune signaling as a key biological domain implicated in disease susceptibility (73). Supporting evidence from murine middle ear transcriptomics and rodent OME models indicates broad activation of TLR- and NLR-related pathways in the middle ear mucosa (74, 75). However, these animal data should be interpreted as mechanistic support rather than direct evidence of human genetic association. Their main relevance in this context is that they strengthen the biological plausibility that innate immune genes identified in human studies may contribute to OM susceptibility by altering pathogen recognition, inflammatory responsiveness, and downstream mucosal defense. Overall, current evidence suggests that dysregulated innate immune recognition and inflammatory control provide a biologically credible pathway linking host genetic variation to OM onset, recurrence, and persistence.

### Epithelial barrier function and Eustachian tube patency

Mucosal epithelial homeostasis and Eustachian tube ventilation are critical to the onset and progression of OM. Genes involved in mucus production, innate epithelial defense, and tissue remodeling, including *MUC5B*, *SFTPD*, and *TGFB1*, have been implicated in middle ear homeostasis and OM susceptibility (59, 76, 77). In particular, *MUC5B* is a major mucin component of chronic middle ear effusions, supporting its relevance to mucus retention and chronic secretory phenotypes. surfactant protein D (SP-D), encoded by *SFTPD*, is present in the Eustachian tube and middle ear and contributes to innate immune defense and disease resolution, whereas polymorphisms in *TGFB1* have been associated with susceptibility to acute otitis media in early infancy. Together, these findings support the view that disruption of epithelial defense, mucus regulation, and tissue remodeling may contribute to persistent or recurrent OM. Moreover, genes involved in ciliary motility, such as *DNAH5* and *DNAI1*, have been well-established contributors to primary ciliary dyskinesia (PCD) (54, 55). In this context, their relevance to OM is biologically plausible, as impaired ciliary motion can promote fluid retention and prolong middle ear inflammation. However, direct evidence that low-penetrance variants in these genes increase OM risk in individuals without classical PCD remains limited.

A key challenge in this area of research is that subtle ciliary variants may be overlooked by routine genetic screening, whereas their contribution to OM severity or chronicity outside recognized ciliopathy syndromes remains unclear. Further large-scale cohort studies are needed to determine the population frequency of such variants and to assess whether mild ciliary dysfunction modifies otitis media severity. Looking ahead, precision interventions for pediatric OM should not only target immune-related pathways but also prioritize the identification of genetic markers associated with Eustachian tube function and epithelial integrity. More broadly, precision approaches for pediatric OM may need to consider not only immune-related pathways but also genetic markers related to Eustachian tube function, epithelial integrity, and mucociliary clearance.

### Oxidative stress damage and environmental response capacity

Oxidative stress acts as a critical intermediary between environmental exposures and host immune responses, playing a central role in the pathogenesis of OM. Upon exposure to infectious agents (e.g., bacterial toxins) or non-infectious stimuli (e.g., air pollution, secondhand smoke), the middle ear mucosal epithelium generates excessive reactive oxygen species (ROS) and reactive nitrogen species (RNS). This oxidative burst leads to lipid peroxidation, DNA damage, and apoptosis, ultimately initiating chronic inflammation, impairing ciliary function, and delaying epithelial repair. Collectively, these changes contribute to the progression from acute to chronic OM (78). The capacity to manage oxidative stress is genetically regulated, with *NFE2L2*, which encodes the transcription factor Nrf2, serving as a master regulator of redox homeostasis in the middle ear. Nrf2 orchestrates the antioxidant response by upregulating downstream enzymes, including HO-1, *SOD2*, and *GPX1*, thereby mitigating ROS-induced cellular injury during infection (64). In a murine OM model, Fan et al. demonstrated that Nrf2 promotes macrophage polarization toward the anti-inflammatory M2 phenotype, facilitating resolution of inflammation and tissue repair while reducing the risk of chronic progression (67). Their population-based study further showed that Nrf2 expression levels increase during disease progression, implicating its role in modulating inflammatory responses by suppressing pro-inflammatory cytokine release (79). Although direct evidence linking *SOD2* and *GPX1* variants to OM susceptibility is lacking, their known roles in free radical scavenging and tissue protection suggest that impaired expression may indirectly contribute to disease pathology. Further high-quality studies are warranted to elucidate their mechanistic involvement in OM. In a cisplatin-induced ototoxicity mouse model, *GSTM1/GSTT1* double knockout resulted in significantly elevated oxidative stress in cochlear tissues. These findings indicate that *GSTM1* deficiency may compromise ROS clearance, thereby exacerbating inflammation and tissue damage in the ear (68). While existing evidence suggests a potential association between *GSTM1* deficiency and increased risk of chronic OM, its exact pathogenic role remains to be fully defined.

These findings underscore the importance of evaluating individual susceptibility to OM, not solely through the lens of infectious exposure but also in the context of host genetic determinants governing antioxidant defense. In particular, the identification of high-risk genotypes associated with the *NFE2L2* signaling pathway or glutathione metabolism may offer a molecular basis for developing future antioxidant-targeted preventive and therapeutic strategies. We propose that the chronicity of OM does not represent a simple linear extension of acute inflammation, but rather reflects a dysregulated interplay between genetic predisposition, environmental exposure, and tissue repair capacity. This imbalance may hinder the timely resolution of inflammation and the restoration of mucosal barrier integrity, thereby exacerbating microbial dysbiosis and epithelial injury. These insights highlight the need for longitudinal cohort studies and mechanistic investigations to further elucidate the gene–exposure–repair network underlying chronic OM.

### Genetic regulation of the nasopharyngeal microbiota

Emerging evidence highlights a dynamic, co-adaptive relationship between the host and its resident microbiota, shaped in part by host genetics. Disruption of key mediators of host–microbiome crosstalk may compromise mucosal homeostasis and alter microbial composition. In the context of OM, *A2ML1* (α-2-macroglobulin-like protein 1) has been identified as a host susceptibility gene encoding a protease inhibitor expressed in the middle ear mucosa. Multiple studies have linked rare repeat variants in *A2ML1* to increased OM susceptibility. For example, an exome sequencing analysis of 243 families demonstrated familial co-segregation of pathogenic or likely pathogenic variants in *A2ML1* and other candidate genes among OM-affected individuals (80). Additional human genetic studies have also identified other mucosal defense–related susceptibility genes, including *SLPI*, supporting the broader relevance of epithelial barrier and immune-regulatory pathways in OM (41). Recently, an *A2ml1* knockout (KO) mouse model generated via CRISPR technology provided the first *in vivo* evidence of its functional role. These mice developed spontaneous OM and exhibited dysregulation of epithelial junctional proteins such as desmoplakin (DSP), implicating *A2ML1* in epithelial adhesion and barrier maintenance during inflammatory responses (69). Collectively, these findings advance our understanding of the genetic regulation of host–microbiome interactions in OM and position *A2ML1* as a key mediator at the interface of epithelial integrity and immune defense.

Another key host susceptibility gene is *FUT2* (Fucosyltransferase 2), which determines secretor status and influences epithelial glycosylation on mucosal surfaces such as the nasopharyngeal epithelium (72). Through this mechanism, functional *FUT2* alleles may shape bacterial adhesion and colonization, whereas loss-of-function variants in non-secretors may increase susceptibility to opportunistic pathogens (72). A multi-ethnic cohort study involving Filipino extended families and U.S. trios identified several *FUT2* loss-of-function variants, including p.Arg202*, p.Trp154*, and p.Arg138Cys, that were significantly associated with recurrent and familial OM; notably, p.Arg202* showed strong co-segregation with OM in Filipino pedigrees (LOD score = 4.0) (72). In addition, a later study showed that the *FUT2* c.461G > A (p. Trp154) variant was associated with differentially expressed genes and nasopharyngeal microbiota shifts in patients with OM (65). Together, these findings support the view that FUT2 may influence OM risk indirectly through modulation of the nasopharyngeal microbial ecosystem (65, 72).

In summary, the identification of host genetic variants such as *A2ML1* and *FUT2* not only elucidates the molecular underpinnings of individual susceptibility to OM but also helps clarify how epithelial integrity, mucosal defense, and microbial ecology intersect in OM pathogenesis. At present, these findings are best viewed as providing a conceptual framework for future risk stratification and microbiome-informed intervention research, rather than as a basis for immediate genotype-guided clinical interventions.

## Environment–host interactions

Although several genetic variants have been linked to increased susceptibility to OM, their effects in children are often modulated by environmental exposures and developmental stage. Rather than acting as isolated determinants, these factors appear to interact through shared inflammatory, oxidative, epithelial, and microbial pathways that together shape disease onset, recurrence, and chronic progression in children (13).

Children often exhibit markedly different clinical outcomes despite similar environmental exposures, reflecting the complex interplay between external factors and intrinsic host biology. From a systems biology perspective, exposure-related signals, microbial ecological shifts, and host inflammatory responses interact within a multidirectional regulatory feedback network. Pollutants such as PM2.5, NO_2_, O_3_, and viral particles can activate the NF-κB signaling pathway, thereby upregulating pro-inflammatory cytokines including IL-6, IL-8, and TNF-α. Concurrently, levels of oxidative stress markers such as 8-hydroxy-2′-deoxyguanosine (8-OHdG) and malondialdehyde (MDA) are elevated. This reflects sustained free radical damage and cellular injury, which contribute to disease progression. Moreover, environmental exposures induce epigenetic modifications, particularly altered promoter methylation in key inflammatory genes such as *TLR4*, *IL6*, and *TNF*, resulting in transcriptional reprogramming of immune responses. For instance, Xie et al. demonstrated that lipooligosaccharide (LOS) derived from *M. catarrhalis* activates human monocytes to produce high levels of IL-8 via a *TLR4*/CD14-dependent pathway, while also inducing peripheral immature monocytes to secrete TNF-α, ultimately driving excessive inflammation in the middle ear mucosa (81). Recent mechanistic studies further suggest that these inflammatory and oxidative pathways are biologically coupled rather than parallel phenomena. In a murine OM model, Nrf2 was shown to regulate the transition from acute to chronic inflammation through macrophage polarization, indicating that antioxidant programs can actively influence inflammatory resolution and tissue repair (67). Consistent with this, pediatric ear-effusion data have shown that Nrf2, *TLR2*, and *TLR4* expression increase with disease severity and correlate positively with inflammatory mediators, supporting co-amplification of oxidative and innate immune signaling in children with more severe OM phenotypes (79). Early-life exposure studies also suggest that pollutant-associated methylation changes may contribute to this interaction framework at the epigenetic level (82). Environmental factors can also influence the composition and diversity of the nasopharyngeal microbiota, thereby indirectly contributing to OM susceptibility. In this context, microbiota alterations may act as an intermediary mechanism linking environmental exposures to host immune responses, pathogen colonization, and disease progression.

Host genetic background appears to modify how these environmental and microbial signals are translated into disease. Variants in pathogen-recognition pathways, particularly *TLR2*- and *TLR4*-related signaling, may influence how microbial or pollutant-derived stimuli are sensed and translated into inflammatory responses (83). Likewise, *FUT2*- and *A2ML1*-related variation may alter epithelial glycosylation, barrier integrity, and colonization dynamics at the mucosal surface (65, 69, 72). In addition, *GSTM1/GSTT1*-related differences in antioxidant defense may affect susceptibility to environmentally induced oxidative injury, although current evidence supports their role more strongly in oxidative injury susceptibility than in directly driving progression from acute to chronic OM (68). Thus, the same environmental exposure may lead to distinct downstream outcomes depending on the host's inflammatory threshold, epithelial resilience, and capacity to maintain microbial homeostasis (101).

**Figure 1:**
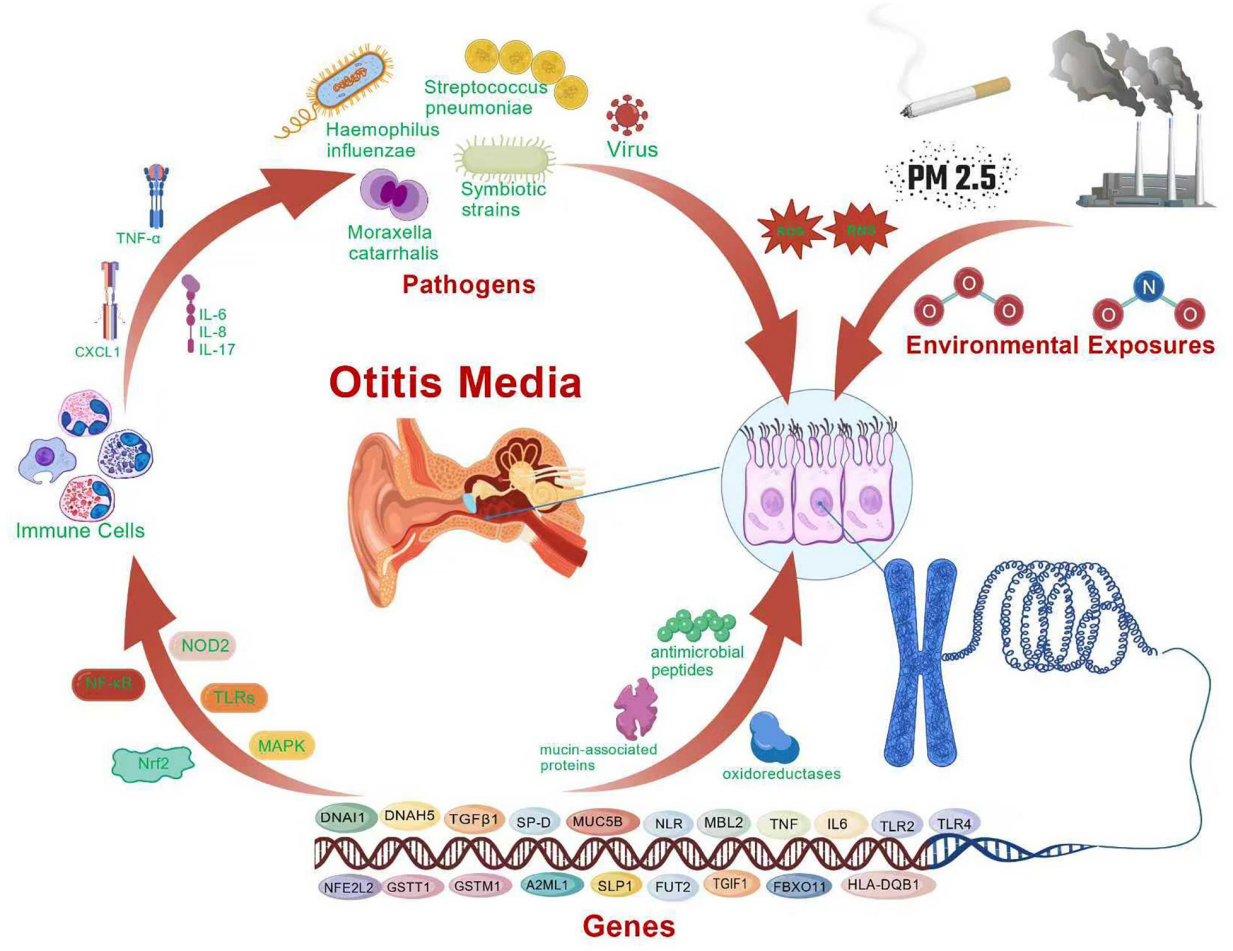
Schematic illustration of the proposed interactions among environmental exposures, microbial dysbiosis, host inflammatory responses, and genetic susceptibility in pediatric otitis media.

Recent systems-level studies further support this interaction framework. Single-cell transcriptomic analysis has shown that the middle ear contains a complex epithelial and immune-cell landscape, with monocytes/macrophages acting as major regulators of the acute response to infection (74). Together, these findings suggest that pediatric OM is shaped by interacting inflammatory, epithelial, oxidative, and microbial-response pathways rather than by isolated single-factor mechanisms. Figure 1 presents a schematic summary of these proposed environment–host–microbiota interactions. In this context, the pathogenesis of pediatric OM may be viewed through an environment–host–microbiota interaction framework, in which environmental exposures, host susceptibility, and microbial dysbiosis interact through interconnected inflammatory and immune pathways (101).

## Biomarkers in pediatric otitis media

The interaction between environmental exposures and host susceptibility in pediatric OM is accompanied by measurable changes in inflammatory and oxidative stress pathways. From a translational perspective, biomarkers may help characterize disease activity, identify children with heightened inflammatory burden, and clarify mechanisms underlying recurrence and chronicity. However, most candidate biomarkers remain exploratory, and their pediatric applicability still depends on stronger validation in age-specific cohorts.

### Inflammation-related markers

In pediatric OM, chronic inflammation can be accompanied by structural changes in the middle ear, with inflammatory processes contributing both to disease pathogenesis and, in some cases, to local tissue damage. Elevated expression of pro-inflammatory cytokines such as tumor necrosis factor-α (TNF-α), interleukin-1α (IL-1α), and interleukin-6 (IL-6) has been reported in cholesteatoma tissue, implicating these mediators in bone resorption processes and chronic inflammatory injury (84, 85). However, most of these data derive from adults or mixed-age populations with chronic OM and bony involvement rather than specifically from children. In children, more recent analyses of ear effusion have shown that inflammatory burden varies with disease severity: TNF-α, interferon-γ (IFN-γ), IL-1β, transforming growth factor-β1 (TGF-β1), and procalcitonin (PCT) were all higher in more severe OM phenotypes, and TNF-α and PCT were significantly higher in children younger than 3 years than in older age groups, suggesting more intense inflammatory activity in early childhood (79). Beyond TNF-α alone, pediatric studies of middle ear effusion have shown that IL-1β, IL-8, and IL-10 are consistently detectable in children with OME, supporting the view that local cytokine profiles may help characterize inflammatory phenotype rather than reflecting a single pro-inflammatory pathway (86, 87). IFN-γ, a key immunomodulatory cytokine, has also been linked to suppurative OM and disease outcome, suggesting that it may reflect persistent mucosal immune activation rather than acute inflammation alone (88). Recent pediatric work has further implicated inflammasome-related mediators, including caspase-1 and IL-18, in OME. Their detection in middle ear effusion suggests that inflammasome activation may represent an additional inflammatory dimension of pediatric OM and may vary with disease duration (89). Markers of systemic inflammation, such as C-reactive protein (CRP), may assist the evaluation of suspected bacterial AOM (90, 91). However, CRP should be regarded as an adjunctive rather than disease-specific biomarker, because viral respiratory infections and post-vaccination inflammatory responses may also elevate CRP levels (92, 93). Thus, relying solely on CRP levels to determine the nature of infection in pediatric OM may lead to overestimation of bacterial disease and inappropriate antimicrobial use.

In addition to cytokines, host-derived local defense molecules may provide complementary biomarker information. In recurrent AOM, high concentrations of antimicrobial peptides and innate defense proteins in middle ear effusion have been associated with pathogen detection, suggesting that local innate immune proteins may reflect persistent bacterial burden and ongoing mucosal immune activation (94). Beyond inflammatory mediators, mucin-related markers may also help characterize effusion phenotype. In chronic middle ear effusions, *MUC5B* appears to be the predominant secretory mucin and may be relevant to effusion viscosity, persistence, and impaired clearance (76). Taken together, these findings suggest that inflammatory biomarkers in pediatric OM may be more informative when interpreted as part of a broader local inflammatory and mucosal response profile rather than as isolated single markers.

### Oxidative stress-related markers

Oxidative stress appears to be an important accompanying process in pediatric OM. Pediatric studies in AOM and otitis media with effusion/chronic otitis media with effusion (OME/COME) have shown increased lipid peroxidation together with altered antioxidant defense, supporting a role for oxidative imbalance in disease activity and chronic progression (64, 95–97). In particular, elevated malondialdehyde (MDA) has been repeatedly interpreted as a marker of cumulative oxidative injury, whereas reduced or altered antioxidant enzyme activities—including superoxide dismutase (SOD), catalase (CAT), and glutathione peroxidase (GPx)—suggest impaired capacity to counterbalance reactive oxygen species in affected children (95–97).

Recent analyses of pediatric ear effusion further suggest that oxidative stress and inflammation are closely linked in OM progression. In children with different OM phenotypes, Nrf2, *TLR2*, and *TLR4* expression in ear effusion increased with disease severity and showed significant positive correlations with inflammatory cytokines, supporting a role for coordinated Nrf2/TLR-related inflammatory–oxidative signaling in chronic progression (79). These findings are consistent with broader OM-focused reviews indicating that oxidative imbalance may contribute to mucosal injury, chronic inflammation, and delayed tissue recovery (64). Overall, MDA and antioxidant enzyme activities such as SOD remain promising exploratory biomarkers for pediatric OM, but their clinical utility remains limited by insufficient pediatric validation, incomplete specificity, and the lack of standardized predictive frameworks. Larger longitudinal studies are still needed before inflammatory and oxidative stress-related biomarkers can be incorporated into clinically actionable monitoring or prediction models in pediatric OM. Representative inflammatory, local innate defense, and oxidative stress-related biomarkers in pediatric otitis media, together with their proposed mechanisms and potential clinical relevance, are summarized in Table 3.

**Table 3:** Mechanisms of action and potential clinical significance of inflammatory, local innate defense, and oxidative stress-related biomarkers in pediatric otitis media.

Category	Biomarkers	Function and mechanism	Clinical relevance	References
Inflammatory cytokines	TNF-α, IL-1α, IL-1β, IL-6, IL-8, IL-10	Regulate local inflammatory responses, mucosal remodeling, and tissue injury; some are associated with bone resorption and chronic inflammatory activity	May help characterize inflammatory phenotype and disease severity, but are not disease-specific	(79, 84–87)
Interferons/inflammasome-related markers	IFN-γ, caspase-1, IL-18	Reflect persistent mucosal immune activation and inflammasome-related inflammatory signaling in middle ear effusion	May help characterize persistent immune activation and an additional inflammatory dimension of pediatric OM, particularly in relation to disease severity or duration	(88, 89)
Acute-phase proteins	CRP	Systemic acute-phase reactant elevated during bacterial infection, but also influenced by viral infection and vaccination-related inflammatory responses	May assist in the evaluation of suspected bacterial acute otitis media, but has limited specificity.	(90–93)
Local mucosal/innate defense markers	Antimicrobial peptides, innate defense proteins, *MUC5B*	Reflect local innate immune activation, pathogen persistence, and effusion phenotype; *MUC5B* is linked to effusion viscosity and impaired clearance	May complement inflammatory biomarkers in identifying persistent bacterial burden and secretory phenotype	(76, 94)
Antioxidant enzymes	SOD, CAT, GPx	Represent endogenous antioxidant defense against reactive oxygen species	May reflect impaired antioxidant capacity in pediatric AOM/OME/COME, but remain exploratory	(95–97)
Oxidative stress markers	MDA	Product of lipid peroxidation; reflects cumulative oxidative injury and tissue damage	May indicate cumulative oxidative injury and support assessment of oxidative imbalance, but remains exploratory	(64, 95–97)

## Microbial factors and microbiota-related signatures

### Microbial ecological alterations and dysbiosis in pediatric OM

In addition to direct pathogen-related mechanisms, accumulating evidence suggests that environmental exposures and clinical interventions, particularly antibiotic use, may substantially reshape the microbial ecosystem. Such perturbations can reduce microbial diversity, promote pathogen dominance, and contribute to dysbiosis, thereby increasing susceptibility to recurrent or chronic otitis media. Antibiotic exposure, commonly used in clinical management, may further contribute to microbial dysbiosis in pediatric OM. Recurrent upper respiratory tract infections in children often result in increased antibiotic use, which can suppress commensal microbiota growth and promote proliferation of antibiotic-resistant bacteria or opportunistic pathogens (98).

Available evidence indicates that in pediatric populations, the predominance of common otopathogens, including *Streptococcus pneumoniae*, *Haemophilus influenzae*, *Moraxella catarrhalis*, and *Staphylococcus aureus*, is shaped by several age-related factors, such as immature immune defenses, frequent viral co-infections, and repeated antibiotic exposure (99, 100). Supporting this view, studies of the upper respiratory microbiota have shown that nasopharyngeal microbial communities undergo dynamic changes during health and illness. In a prospective cohort including 1,616 healthy children and 1,423 children with respiratory infections, marked differences in nasopharyngeal microbiota composition were observed between groups, and approximately 91% of samples were dominated by profiles centered on *Haemophilus*, *Streptococcus*, *Moraxella*, and *Staphylococcus* (101, 102).

These findings suggest that microbial community structure in the upper airway is highly dynamic and may be influenced by both host factors and environmental exposures. Environmental exposures may further exacerbate these alterations by reducing microbial diversity and facilitating pathogen dominance, thereby contributing to dysbiosis and increasing susceptibility to infection. A case-control study by Lappan et al. found a marked depletion of potentially protective genera, such as *Dolosigranulum* and *Corynebacterium*, in the nasopharynx of children with recurrent AOM, highlighting the role of microbial imbalance in disease susceptibility (103). More recent work suggests that microbial dysbiosis is not merely a downstream consequence of exposure or host vulnerability, but may itself act as an active intermediary that amplifies disease through enhanced pathogen persistence and biofilm-associated chronicity. In an otitis media standard-of-care model, broad-spectrum antibiotics induced substantial fecal and nasopharyngeal microbiome disruption, illustrating how treatment-related perturbation may further destabilize microbial ecology (104). Recent pediatric OM microbiome reviews have likewise emphasized the mutually reinforcing relationship between dysbiosis and biofilm formation in driving persistence, immune evasion, and transition from acute to recurrent or chronic disease (105). Age-dependent studies of adenoid microbiota further suggest that these microbial effects may vary across childhood rather than remaining biologically static (106).

Taken together, these findings indicate that environmentally driven microbial dysbiosis may act as a key intermediary linking external exposures to disease susceptibility. Consistent with these mechanistic insights, lower microbial diversity and pathogen-enriched profiles were associated with greater susceptibility to OM. In parallel, studies comparing nasopharyngeal and middle ear microbiota in children with AOM, together with case-control data in recurrent AOM, suggest that dysbiosis and depletion of potentially protective genera such as *Dolosigranulum* and *Corynebacterium* may facilitate pathogen translocation to the middle ear, particularly in the setting of viral co-infection, thereby increasing the risk of recurrent or persistent disease (103, 107).

### Pathogen-related marker genes

Several pathogen-related marker genes have been implicated in the virulence, persistence, and clinical heterogeneity of pediatric OM. In *S. pneumoniae*, *ply* and *lytA* are linked to inflammatory tissue injury, autolysis, and release of virulence factors in experimental otitis media models, whereas *luxS* contributes to quorum sensing and biofilm-associated persistence (108–110). In *H. influenzae*, *hpd*, which encodes outer membrane protein D, has been studied as an adhesion- and vaccine-related marker, while *fucK* has been associated with metabolic adaptation and persistence in the airway environment (111, 112). In *M. catarrhalis*, *ompP2* is related to outer membrane permeability and antimicrobial susceptibility, whereas *uspA1* plays an important role in epithelial adhesion and invasion, supporting nasopharyngeal colonization and ascending infection (113–115). In addition, in *S. aureus* and *Streptococcus pyogenes*, *mecA*, *icaA*, and *speB* are associated with methicillin resistance, biofilm-related persistence, and immune evasion, respectively, thereby increasing treatment complexity in chronic or suppurative disease (116–118). Representative pathogen-related marker genes and their proposed functional relevance in pediatric OM are summarized in Table 4.

**Table 4:** Pathogenic bacteria associated with pediatric otitis media and the functions of their marker genes.

Bacterium	Marker gene	Functional description	Disease relevance	Reference
*Streptococcus pneumoniae*	*ply*	Encodes pneumolysin (Ply), a cytotoxic hemolysin that activates the complement system and enhances inflammatory responses.	Closely associated with the onset of AOM, promoting inflammation and tissue damage.	(108)
	*lytA*	Encodes autolysin LytA, involved in cell wall degradation and autolysis, facilitating the release of virulence factors.	Upregulated during invasive infections; enhances bacterial invasiveness and virulence.	(109)
	*luxS*	Participates in quorum sensing; regulates biofilm formation and expression of resistance genes.	Contributes to persistent infections and antibiotic tolerance in chronic otitis media.	(110)
*Haemophilus influenzae*	*hpd*	Encodes outer membrane protein D, involved in adhesion and biofilm formation.	Strongly associated with the development of OME, a potential vaccine target.	(111)
	*fucK*	Involved in L-fucose metabolism, providing energy and carbon sources.	Enhances environmental adaptability; may support metabolic adaptation and persistence in the airway environment.	(112)
*Moraxella catarrhalis*	*ompP2*	Encodes outer membrane protein P2, forms porins, and influences molecular permeability.	Closely related to β-lactam resistance, which affects treatment efficacy.	(113)
	*uspA1*	Encodes surface protein UspA1, promotes bacterial adhesion, and immune evasion.	Plays a key role in nasopharyngeal colonization and ascending infections, increasing infection risk.	(114, 115)
*Staphylococcus aureus*	*mecA*	Encodes PBP2a, conferring resistance to methicillin and other β-lactam antibiotics.	Frequently detected in MRSA-associated otitis media, it increases treatment complexity.	(116)
	*icaA*	Involved in the synthesis of polysaccharide intercellular adhesin, promoting biofilm formation.	Associated with chronic and recurrent infections and biofilm-related antibiotic resistance.	(117)
*Streptococcus pyogenes*	*speB*	Encodes cysteine protease SpeB, which degrades host tissues and immune components.	Can cause acute suppurative otitis media; facilitates tissue damage and immune evasion.	(118)

### Antimicrobial resistance genes and mechanisms

Antimicrobial resistance is an increasingly important determinant of treatment failure, persistence, and recurrence in pediatric OM. In pneumococci and streptococci, macrolide resistance is frequently associated with *ermB*-mediated 23S rRNA methylation and *mefA*-related efflux mechanisms, while quinolone resistance may arise through *gyrA/parC* mutations (119–124). Resistance to β-lactam antibiotics in *S. pneumoniae* is commonly linked to alterations in *pbp1a/pbp2b/pbp2x*, which reduce the affinity of penicillin-binding proteins for penicillins and cephalosporins (125, 126). In *S. aureus*, additional resistance determinants include *mupA*, which mediates mupirocin resistance, whereas *vanA/vanB* remain clinically important in enterococci because of their relevance to severe vancomycin-resistant infections (127–130). Among otopathogens, *H. influenzae* frequently carries *blaTEM* and *blaROB*, which encode *TEM-1* and *ROB-1* β-lactamases, respectively (131–134), whereas *M. catarrhalis* characteristically produces the BRO-type β-lactamases BRO-1/BRO-2 rather than TEM-type enzymes (135, 136). Chloramphenicol resistance may be mediated by *cat*, and tetracycline resistance by *tetM*, further illustrating the diversity of resistance mechanisms that may complicate antimicrobial management in OM-related infections (137–140). Representative antimicrobial resistance genes, their mechanisms, and their potential clinical implications are summarized in Table 5.

**Table 5:** Bacterial resistance mechanisms and genes associated with pediatric otitis media.

Category	Resistance gene	Bacteria	Mechanism	Clinical impact	References
Target modification	*ermB*	*S. pneumoniae*, *S. pyogenes*	Methylation of 23S rRNA, blocking macrolide binding	Confers resistance to macrolides (e.g., erythromycin, azithromycin) but remains sensitive to β-lactams	(121, 122)
	*gyrA/parC*	*S. pneumoniae*, *S. aureus*	Mutations in DNA gyrase/topoisomerase	Confers resistance to fluoroquinolones (e.g., levofloxacin)	(119, 120)
	*pbp1a/pbp2b/pbp2x*	*S. pneumoniae*	Altered penicillin-binding proteins reduce β-lactam affinity	Increased resistance to penicillins and cephalosporins	(125, 126)
	*mupA*	*S. aureus*	Mutations in isoleucyl-tRNA synthetase	Resistance to mupirocin; affects topical treatment in skin and nasal infections	(127, 128)
	*vanA/vanB*	*Enterococcus* spp.	Alteration in peptidoglycan precursor reduces vancomycin binding	Leads to vancomycin-resistant enterococci (VRE), common in severe infections	(129, 130)
Antibiotic inactivation	*blaTEM*	*H. influenzae*	Production of TEM-1 β-lactamase that hydrolyzes aminopenicillins	Confers resistance to ampicillin/amoxicillin; amoxicillin-clavulanate usually remains effective	(131, 132)
	*blaROB*	*H. influenzae*	Production of ROB-1 β-lactamase	May reduce susceptibility to cefaclor and cefprozil; β-lactam/β-lactamase inhibitor combinations usually remain active	(133, 134)
	*BRO-1/BRO-2*	*M. catarrhalis*	Production of BRO-type β-lactamases	Confers resistance to penicillin/ampicillin and affects β-lactam susceptibility	(135, 136)
	*cat*	*S. pneumoniae*, *S. aureus*	Chloramphenicol acetyltransferase inactivates the drug	Resistance to chloramphenicol (less commonly used in pediatric otitis media)	(137, 138)
Efflux pumps	*mefA*	*S. pneumoniae*	Encodes an efflux pump that lowers intracellular macrolide concentrations	Confers erythromycin resistance; clindamycin may still be effective	(123, 124)
Ribosomal protection	*tetM*	*S. pneumoniae*, *Enterococcus*	Ribosomal protection protein prevents tetracycline binding	Resistance to tetracyclines (e.g., doxycycline)	(139, 140)

In summary, microbial virulence-associated markers and antimicrobial resistance determinants broaden our understanding of pediatric OM beyond simple pathogen identification, emphasizing that microbial composition, biofilm-related persistence, and resistance traits are integral components of the broader environment–host–microbiome framework underlying disease recurrence and chronicity.

## Conclusion

In summary, environmental exposures and host genetic susceptibility together with microbial ecology jointly shape susceptibility to the onset, recurrence, and progression of pediatric OM. Due to the immaturity of the Eustachian tube and the developing immune system, children are particularly vulnerable to external stimuli such as air pollutants, allergens, and pathogenic microorganisms. Environmental factors can compromise epithelial barrier integrity and impair mucociliary clearance, while also triggering local immune and inflammatory responses, as well as oxidative imbalance, thereby substantially increasing the risk of OM. Genetic variations, especially polymorphisms in genes involved in immune regulation and mucosal defense, further heighten individual sensitivity to environmental insults and pathological stimuli. The interplay between genetic predisposition and environmental exposure contributes to the clinical and mechanistic heterogeneity of pediatric OM, promoting recurrent episodes, chronic disease progression, and associated complications. Moreover, the nasopharyngeal microbiome serves as an important intermediary in this complex interaction. Alterations in microbial community composition and function, shaped by both environmental factors and host genetics, may mediate susceptibility to OM and influence disease outcomes. At the same time, emerging evidence on inflammatory mediators, oxidative stress-related indicators, local mucosal defense molecules, pathogen-related marker genes, and antimicrobial resistance determinants has added further resolution to the biological heterogeneity and therapeutic complexity of pediatric OM. With the advent of multi-omics technologies, a growing body of research is focusing on how environmental exposures modulate immune development via epigenetic regulation and microbiome dysbiosis. Comprehensive analysis of this intricate network, which encompasses environmental, genetic, and microbial factors, particularly during critical developmental windows, holds promise for identifying high-risk individuals and elucidating precise pathogenic mechanisms. Such efforts will be essential for advancing early diagnosis, future risk stratification, and the development of targeted interventions in pediatric OM.

### Limitations and future prospects

Although current research has preliminarily established a multidimensional interactive framework for pediatric OM based on the “environment–genetics–microbiome” model, significant limitations remain in mechanism integration, translational application, and population-specific studies. Most existing studies focus on correlation analyses of isolated factors and lack causal inference supported by multi-omics integration and functional validation. Given the complex etiology of OM, there is an urgent need to construct a comprehensive pathogenic network incorporating genomic, epigenomic, and metagenomic data. Studies investigating exposure–response relationships during the critical developmental window of early childhood (ages 0–5) are particularly scarce. However, both middle ear and immune system development exhibit pronounced age-dependent characteristics. This necessitates dynamic mechanistic studies using longitudinal pediatric cohorts with repeated sampling and more precise exposure assessment. In this context, keratin-based matrices such as hair and nails may serve as useful non-invasive tools for selected longer-term exposure assessments in children. For example, a study in school-aged children showed that hair and toenail manganese concentrations were associated with manganese exposure from drinking water, supporting the value of keratinized tissues as indicators of cumulative environmental exposure (141). In addition, studies of prenatal tobacco exposure have shown that maternal and infant hair and nail nicotine levels can reflect intrauterine smoke exposure, indicating that these matrices may also be informative for early-life exposure assessment (142). More broadly, hair and nails have been explored as integrated biomarkers of persistent environmental contaminants, including flame retardants, because they can provide cumulative exposure information beyond short-term blood or urine measurements (143). Nevertheless, the application of these matrices still requires exposure-specific validation and methodological standardization, particularly with respect to external contamination, sample preparation, and analyte-specific interpretation. The clinical application of biomarkers also remains limited. Most candidate molecules are still at an exploratory stage and lack validation in large pediatric cohorts, as well as standardized protocols for predictive model development and external validation. In addition, antimicrobial resistance research has largely focused on isolated clinical strains, with insufficient monitoring of resistance gene transmission within community networks. Globally, especially in low- and middle-income regions, public health interventions for pediatric OM remain fragmented, and the disease burden remains high. There is a pressing need for integrated strategies combining air pollution control, vaccination programs, and health education tailored for children.

In terms of innovative approaches, research is shifting toward multi-omics integration, AI-assisted predictive modeling, and cross-sector public health collaboration. Multi-layered analyses of the genome, epigenome, and microbiome can uncover regulatory mechanisms by which environmental exposures influence OM susceptibility. For example, GWAS have identified immune-related genes such as *TLR4* and *MBL2*, while epigenetic research has shown that pollutants like NO_2_ can induce DNA methylation changes affecting immune pathways. Microbiome studies have revealed that antibiotic use can disrupt nasopharyngeal microbial ecology, leading to microbial imbalance, persistence of otopathogens, and potential increases in treatment difficulty. These findings highlight the need for microbiome-targeted therapeutic strategies. AI and machine learning offer significant potential for integrating multidimensional data, enabling personalized risk prediction and biomarker discovery. For instance, combining data on air pollution, genetic variants, and microbial profiles can support the development of predictive models to inform treatment decisions and improve the accuracy of automated otoscope image interpretation. For clinical translation, a risk-based stratification system may represent a useful future framework rather than an established management strategy in pediatric OM (144, 145). Current evidence suggests that such models would most plausibly integrate clinical history, recurrence pattern, and established environmental risk factors, whereas the added value of genetic and microbial profiling still requires prospective validation (144, 145). Current practice guidelines continue to emphasize clinical diagnosis, observation, follow-up, and risk-factor management, rather than routine omics-based stratification or microbiome surveillance in asymptomatic children (145). Likewise, microbiome-informed strategies and immune-directed interventions remain promising but exploratory, and evidence for their preventive use in routine pediatric care remains limited or inconsistent (146, 147). Looking forward, advancing OM prevention and treatment will require multidisciplinary collaboration across genetics, environmental health, AI, and epidemiology. The goal is to transition from descriptive studies to an integrated precision medicine paradigm—one that combines mechanistic insights, predictive modeling, and targeted interventions to enable early detection, precise prevention, and individualized management of pediatric otitis media.
